# AGASI: A Generative Adversarial Network-Based Approach to Strengthening Adversarial Image Steganography

**DOI:** 10.3390/e27030282

**Published:** 2025-03-09

**Authors:** Haiju Fan, Changyuan Jin, Ming Li

**Affiliations:** 1 College of Computer and Information Engineering, Henan Normal University, Xinxiang 453007, China; fanhaiiju8706@163.com (H.F.); liming@htu.edu.cn (M.L.); 2 Henan Provincial Key Laboratory of Educational Artificial Intelligence and Personalized Learning, Xinxiang 453007, China

**Keywords:** steganography, adversarial attacks, generative adversarial network (GAN), information security

## Abstract

Steganography has been widely used in the field of image privacy protection. However, with the advancement of steganalysis techniques, deep learning-based models are now capable of accurately detecting modifications in stego-images, posing a significant threat to traditional steganography. To address this, we propose AGASI, a GAN-based approach for strengthening adversarial image steganography. This method employs an encoder as the generator in conjunction with a discriminator to form a generative adversarial network (GAN), thereby enhancing the robustness of stego-images against steganalysis tools. Additionally, the GAN framework reduces the gap between the original secret image and the extracted image, while the decoder effectively extracts the secret image from the stego-image, achieving the goal of image privacy protection. Experimental results demonstrate that the AGASI method not only ensures high-quality secret images but also effectively reduces the accuracy of neural network classifiers, inducing misclassifications and significantly increasing the embedding capacity of the steganography system. For instance, under PGD attack, the adversarial stego-images generated by the GAN, at higher disturbance levels, successfully maintain the quality of the secret image while achieving an 84.73% misclassification rate in neural network detection. Compared to images with the same visual quality, our method increased the misclassification rate by 23.31%.

## 1. Introduction

Steganography [1] is a key technology in the field of information security, which allows secret data to be hidden in seemingly innocuous media. It has a wide range of applications in secure communication, copyright protection, and other fields. However, existing steganographic methods [2,3] have significant limitations. Ref. [3] mainly discusses the drawbacks of the Least Significant Bit (LSB)-based steganographic method. This method hides secret information by modifying the least significant bit of an image. However, this approach has the following limitations: Since LSB steganography only modifies the least significant bit of an image, the amount of information it can hide is relatively small. Although the capacity can be improved through modifications, there is still a trade-off between capacity and image quality. When embedding secret information, especially in applications where image quality is critical, LSB steganography can lead to significant degradation in image quality, affecting the visual appearance of the image. The limitations mainly include weak resistance to steganalysis, difficulties in extracting steganographic information, and vulnerability to increasingly sophisticated detection methods. Specifically, the ability of neural network classifiers to identify stego-images has continuously improved, making it more difficult to hide steganographic data. Furthermore, with the introduction of adversarial perturbations, the hidden secret information (such as text, images, videos, etc.) becomes increasingly difficult to extract. These factors collectively present significant challenges to the security of information-hiding technologies [4]. Therefore, enhancing the resistance of stego-images to steganalysis while improving the accuracy of secret information extraction is a key research direction for advancing steganography.

To address these challenges, researchers have increasingly explored the introduction of adversarial perturbations in image processing to enhance privacy protection. Yuan et al. [5] reviewed the application of adversarial training methods in defending against adversarial attacks, discussed various adversarial sample generation techniques and their impact on model robustness, and analyzed current challenges and future research directions. Jia et al. [6] combined image watermarking techniques with adversarial algorithms, proposing a visual watermark perturbation algorithm that uses the BHE optimization algorithm to determine the embedding location of the watermark in the image, misleading the output of classification models and thereby achieving privacy and copyright protection. Additionally, generative adversarial networks (GANs) have gained widespread attention in the fields of image generation and processing. Through adversarial learning between a generator and a discriminator, GANs effectively generate high-quality images [7]. This method has not only been successful in image synthesis but also shows great potential in generating adversarial samples. Studies have shown that adversarial samples generated by GANs can significantly enhance the robustness and performance of deep learning models [8,9], improving the models’ resistance to adversarial attacks [10]. The application of GANs in adversarial sample generation has greatly advanced the field of adversarial learning [11]. In the context of steganography methods that resist adversarial steganalysis, Liu et al. [12] primarily explored how to improve steganographic security by generating and selecting stego-images. Ghamizi et al. [13] proposed a method of adversarial steganography that uses the target attack label as secret information, transmitting the secret information through the generated adversarial image’s class label. However, the existing research still faces some limitations: on the one hand, it either sacrifices image quality to improve detection error rates, or it reduces the amount of secret information embedded to improve image quality; on the other hand, the embedded watermark may be too conspicuous, attracting attention from third parties.

To address these issues, this paper proposes AGASI, a method that significantly improves the error detection rate of neural network classifiers while maintaining the quality and embedding capacity of secret information. Specifically, AGASI enhances the adversarial nature of images by introducing generative adversarial networks (GANs), effectively protecting stego-images from deep learning-based analysis attacks, while also improving the security of steganography. The main contributions of this paper are as follows:

- The encoder functions as the generator in the generative adversarial network (GAN), introducing adversarial learning to minimize the discrepancy between the extracted secret information and the original secret information.
- The adversarial stego-images induce a higher error classification rate in neural networks, while the secret information extracted by the decoder maintains high quality.
- Under stronger adversarial perturbations, the AGASI method results in images with higher information entropy, embedding more information.

## 2. Related Work

### 2.1. Steganography Methods

Steganography aims to conceal secret information within cover images and has been an area of research for many years. Traditional methods, such as Least Significant Bit (LSB) steganography [14], are widely used due to their simplicity, but these methods are highly vulnerable to steganalysis and can be easily detected. To enhance the robustness of steganography, researchers have proposed various advanced techniques. For example, adaptive steganography improves security by adjusting the embedding process based on the properties of the cover image, while multi-carrier steganography increases information capacity and concealment by embedding data across multiple domains (such as spatial and frequency domains).

In recent years, researchers have focused on the inherent characteristics of images, such as edges and textures, to optimize the embedding process. For example, by using the Kirsch edge detector [15], secret information can be embedded in the edge regions of an image, making it less noticeable and thereby enhancing both the concealment and capacity of steganography. Liu et al. [16] proposed a method that decomposes images into structural and textural components, transforming the secret information into a composite structural image, and significantly improving extraction accuracy by incorporating texture information into the structural image.

With the advancement of deep learning, significant progress has been made in the field of steganography, with many deep learning-based methods outperforming traditional methods in terms of security, concealment, and capacity. Baluja et al. [17] proposed a deep neural network-based framework for steganography that utilizes a preprocessing network, hidden network, and revealing network to hide secret images within cover images. Pan et al. [18] applied deep reinforcement learning to dynamically select the most suitable regions for embedding, converting secret images into encrypted noise and embedding them into local regions of the cover image, thereby enhancing both the security and capacity of the steganography.

In recent years, generative adversarial networks (GANs) have emerged as a promising solution in the field of steganography due to their ability to generate realistic images. A GAN consists of two networks—the generator and the discriminator—which work together to enhance the quality of the stego-images while preserving the secrecy of the embedded information. Wang et al. [19] proposed a GAN-based steganographic algorithm that embeds binary secret information into images, achieving a high embedding capacity of 4.4 bits per pixel (bpp). Yang et al. [20] utilized GANs to design a loss function model that optimizes the steganographic process, reduces the training time, and improves the effectiveness of embedding.

Additionally, adversarial examples have become a key research direction in evaluating the security and robustness of machine learning models. Adversarial examples deceive classifiers by adding tiny, visually imperceptible perturbations to input data. In the field of steganography, adversarial examples present a novel approach to simultaneously conceal information and deceive target classifiers, a technique known as adversarial steganography. Zhang et al. [21] proposed a method that transforms secret information into adversarial perturbations and embeds them into cover images, generating stego-images that not only conceal secret information but also successfully evade adversarial detection. Ghamizi et al. [22] designed a steganographic algorithm based on evasion attacks, using the binary data of the secret information as the attack target to generate adversarial examples. Experiments demonstrate that this method exhibits strong robustness.

Further research, such as the work by Tang et al. [23], combines adversarial examples with steganographic embedding algorithms, adjusting perturbations based on the backpropagation gradient of the steganalysis model, thereby minimizing the probability of detection. Experimental results demonstrate that this approach effectively evades detection by steganalysis models, thereby improving the resistance of steganography to both adversarial examples and steganalysis. Recent research has been significantly influenced by deep learning and GAN techniques, greatly enhancing the concealment and security of hidden information. Simultaneously, the integration of adversarial machine learning techniques has opened new research directions, leading to the development of stego-images capable of resisting both steganalysis and adversarial attacks, thereby providing new frontiers for information-hiding research.

### 2.2. Steganalysis Methods

Steganalysis is the process of detecting hidden secret information within images, and its effectiveness has been significantly enhanced by the application of deep learning techniques, particularly in the field of image processing. In recent years, steganalysis methods based on convolutional neural networks (CNNs) have made remarkable progress, especially with the development of efficient network architectures such as DenseNet-40 and Wide ResNet, which have greatly improved the capabilities of steganalysis systems. Huang et al. [24] pointed out that dense connections not only improve information flow efficiency but also reduce the number of parameters, thus enhancing the model’s overall efficiency. Studies applying DenseNet-40 to steganalysis tasks have demonstrated that its detection performance surpasses traditional CNN architectures across multiple datasets, particularly when detecting GAN-generated steganographic images, where DenseNet-40 continues to maintain a high accuracy. This characteristic makes DenseNet a powerful tool in steganalysis, providing robust resistance against various steganographic techniques.

Zagoruyko and Komodakis [25] demonstrated that increasing the network width significantly enhances network performance. In the context of steganalysis, Wide ResNet excels at extracting richer features from different scales and complex patterns when processing steganographic images. Its superior performance has made Wide ResNet a widely adopted model in modern steganalysis tasks, particularly in handling adversarial and high-complexity steganographic images, where it showcases remarkable feature-learning capabilities. Moreover, several studies have shown that other architectures, such as ResNet and VGG, have also been successfully applied to steganalysis tasks. However, the combination of wider and deeper architectures, such as Wide ResNet, has proven to be a game changer for detecting increasingly sophisticated hidden patterns in steganographic images. These models have proven highly effective in detecting subtle perturbations caused by adversarial attacks, which are becoming more prevalent as evasion techniques continue to improve.

Additionally, more recent work by Li et al. [26] demonstrated that combining ensemble learning methods with CNN-based steganalysis models could further enhance the robustness of detection systems. This approach aggregates the predictions of multiple models to generate a more reliable and accurate final output, significantly improving the performance in detecting diverse steganographic techniques across various datasets. The integration of multiple model outputs has shown promise in boosting the detection accuracy for complex and adversarial steganographic images. Similarly, Zhang et al. [27] explored the use of generative models, such as generative adversarial networks (GANs), in conjunction with CNNs to enhance the performance of steganalysis systems. GANs are increasingly utilized to generate adversarial steganographic images, which present an additional challenge for detection models and provide an opportunity to train more resilient steganalysis methods. By focusing on both high-quality feature extraction and adversarial robustness, these modern CNN architectures, including DenseNet-40 and Wide ResNet, continue to push the boundaries of steganalysis technology.

## 3. Our Approach

### 3.1. AGASI Framework Overview

To ensure the privacy and security of the secret image as well as the quality of the extracted image, we propose AGASI (A GAN-Based Approach for Strengthening Adversarial Image Steganography). This method comprises three stages: the stego-image generation and extraction stage, the adversarial stego-image generation stage, and the training stage of the generative adversarial network (GAN).

**Figure 1:**
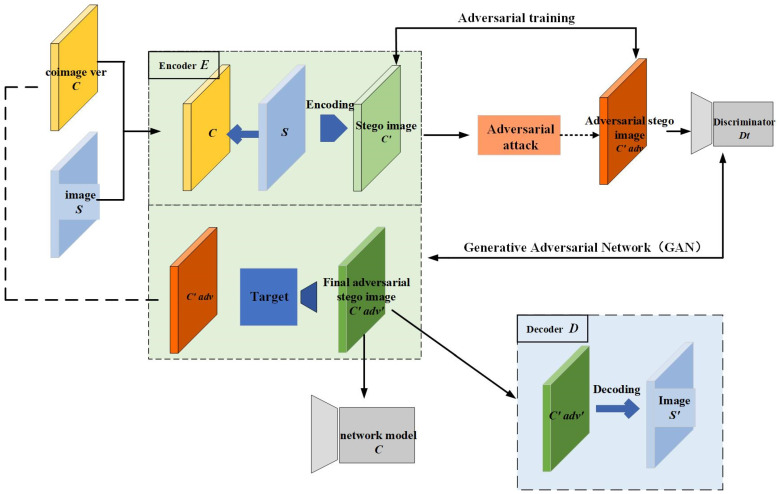
Framework of AGASI: The encoder embeds the secret grayscale image (S) into a color cover image (C), generating a stego-image ($C^{\prime }$). Next, the stego-image ($C^{\prime }$) undergoes adversarial perturbation attacks, and through adversarial training, an adversarial stego-image (${C^{\prime }}_{adv}$) is generated. Subsequently, the encoder and discriminator form a generative adversarial network (GAN), where the discriminator’s task is to distinguish between the cover image (C) and the adversarial stego-image (${C^{\prime }}_{adv}$), ultimately generating the adversarial stego-image (${C^{\prime }}_{adv^{\prime }}$). Finally, after processing through the decoder, the secret grayscale image ($S^{\prime }$) is successfully extracted from (${C^{\prime }}_{adv^{\prime }}$).

AGASI combines adversarial training with generative adversarial networks (GANs), consisting of a generator, discriminator, decoder, and target classifier. By training the GAN, AGASI not only enhances the quality of the stego-images but also effectively safeguards the embedded secret information from third-party attacks. The workflow of the entire method is illustrated in Figure 1.

### 3.2. Encoder and Decoder

The encoder and decoder are key components of the steganography model, and their performance directly impacts the quality of the stego-image and the accuracy of the extracted secret information. The encoder in this study is based on the initial structure from [28], with optimizations and extensions to enhance its performance. The first layer of the encoder is ConvolutionBlock1, which includes a 3 × 3 convolution, batch normalization (BN), and LeakyReLU, used for the initial extraction of image features. The second to eighth layers utilize the Inception module, which extracts multi-scale features through multiple parallel convolutional branches, significantly enhancing the feature extraction capability of the encoder. Specifically, the Inception module consists of the following four branches:

- Branch 1: Performs 1 × 1 convolution + BN + ReLU, followed by 3 × 3 convolution.
- Branch 2: Performs 1 × 1 convolution + BN + ReLU, followed by 5 × 5 convolution.
- Branch 3: Contains 1 × 1 convolution + BN + ReLU, followed by 5 × 5 convolution.
- Branch 4: Uses 3 × 3 max pooling, followed by 1 × 1 convolution + BN + ReLU.

Compared to the single convolutional layer design in the initial structure, the encoder in this study processes multiple scales of features in parallel, allowing it to better capture the complexity and detailed information of the image, thus improving the quality and imperceptibility of the stego-image. The ninth layer is ConvolutionBlock1, which includes 3 × 3 convolution, batch normalization (BN), and LeakyReLU, used for further integration and refinement of features. The output layer uses ConvolutionBlock2 (1 × 1 convolution + Tanh), generating the stego-image.

**Figure 2:**
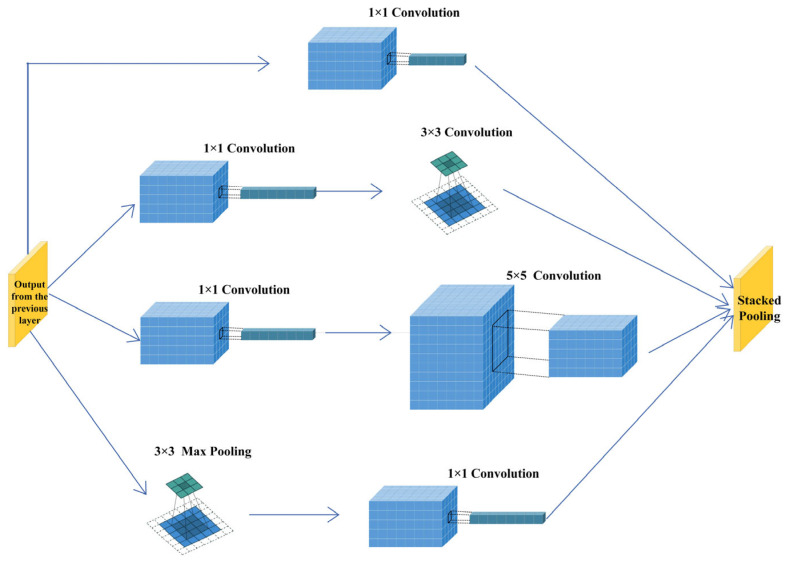
The Inception module processes the input image in parallel using convolutional kernels and pooling operations of various sizes, enabling it to capture features at different scales, thereby enhancing the network’s expressiveness and efficiency.

The decoder network consists of five layers of ConvolutionBlock1, each including 3 × 3 convolution, batch normalization (BN), and LeakyReLU. These layers progressively decode the stego-information and restore the original secret image layer by layer. The output layer of the decoder is ConvolutionBlock2, using 1 × 1 convolution and the Tanh activation function to ultimately extract the secret image. The decoder design in this study remains consistent with the initial structure [28], but optimizations have been made in terms of network depth and the specific design of the convolutional blocks to better restore the stego-information. The process of the Inception module is illustrated in Figure 2.

For the loss function, the Mean Squared Error (MSE) calculates the average of the squared differences between the pixel values of the original image and the processed image. H and W represent the number of rows and columns of the image, while x(i,j) and y(i,j) represent the true value and predicted value at the position (i,j), respectively. The specific form is given by Equation (1).

(1) $$MSE=\frac{1}{HW}\sum_{i=1}^{H}\sum_{j=1}^{W}{\left(x(i,j)-y(i,j)\right)}^{2}$$

The Structural Similarity Index (SSIM) [29] not only considers pixel-level errors but also evaluates the structural information of the image (such as luminance, contrast, and structure), which aligns more closely with human visual perception. It captures both local details and the overall structure of the image. This process is represented by the following Equations (2)–(5):

(2) SSIM(x,y)=L(x,y)C(x,y)S(x,y)

(3) $$L(x,y)=\frac{2\mu_{x}\mu_{y}+c_{1}}{\mu_{x}^{2}+\mu_{y}^{2}+c_{1}}$$

(4) $$C(x,y)=\frac{2\sigma_{x}\sigma_{y}+c_{2}}{\sigma_{x}^{2}+\sigma_{y}^{2}+c_{2}}$$

(5) $$S(x,y)=\frac{\sigma_{xy}+c_{3}}{\sigma_{x}+\sigma_{y}+c_{3}}$$

SSIM(x,y) represents the Structural Similarity Index between two images x and y. L(x,y), C(x,y), and S(x,y) measure the similarity in luminance, contrast, and structure, respectively. μ and σ represent the correlation in terms of average luminance, luminance standard deviation, and luminance variation, respectively, while c is a constant used to avoid division by 0.

Multiscale Structural Similarity (MS-SSIM) [30] evaluates the structural similarity of images at different scales, capturing both the details and global structure of the image at various resolutions, providing a more comprehensive quality assessment. It is specifically represented by Equation (6):

(6) $$MSSSIM(x,y)={\left[L_{N}(x,y)\right]}^{l_{N}}\prod_{j=1}^{N}[C_{j}{\left(x,y\right)}^{y_{j}}][S_{j}{\left(x,y\right)}^{\rho_{j}}]$$

where $L_{N}(x,y)$ represents the computed result of the luminance component at the N-th scale, while $l_{N}$ controls the influence of luminance on the final MS-SSIM. $\rho_{j}$ controls the influence of the structure at this scale on the final MS-SSIM. $\prod_{j=1}^{N}$ represents the combination of the contrast and structural components across N scales. Based on these three metrics, the loss function of the encoder can be expressed by Equation (7).

(7) $${\mathrm{Loss}}_{\mathrm{encoder}}(x,y)=\alpha \cdot (1-L_{N}(x,y))+\beta \cdot \left(1-\prod_{j=1}^{N}C_{j}{\left(x,y\right)}^{y_{j}}\right)+\gamma \cdot \left(1-\prod_{j=1}^{N}S_{j}{\left(x,y\right)}^{\rho_{j}}\right)$$

The loss function of the decoder can be defined by Equation (8).

(8) $${\mathrm{Loss}}_{\mathrm{decoder}}(x,y)=\alpha^{\prime }\cdot (1-L_{N}(y,z))+\beta^{\prime }\cdot \left(1-\prod_{j=1}^{N}C_{j}{\left(y,z\right)}^{y_{j}}\right)+\gamma^{\prime }\cdot \left(1-\prod_{j=1}^{N}S_{j}{\left(y,z\right)}^{\rho_{j}}\right)$$

The total loss of the encoder and decoder can be expressed by Equation (9).

(9) $$\mathrm{TotalLoss}(x,y,z)={\mathrm{Loss}}_{\mathrm{encoder}}(x,y)+{\mathrm{Loss}}_{\mathrm{decoder}}(y,z)$$

### 3.3. Method for Generating Adversarial Stego-Images

The role of the adversarial stego-image (${C^{\prime }}_{adv}$) is to reduce the accuracy of the neural network analyzer, preventing it from detecting the category of the cover image, thereby protecting the secret image. To preserve the quality of the extracted secret image, the adversarial attack is applied to the high-frequency region formed by the Discrete Cosine Transform (DCT) of JPEG images, minimizing the impact on the visual quality of the image [31]. After the encoder encodes the cover image (C) and secret image (S) into a stego-image, the stego-image undergoes attacks using the Fast Gradient Sign Method (FGSM) [32] and Projected Gradient Descent (PGD) [33], generating the adversarial stego-image (${C^{\prime }}_{adv}$), which reduces the accuracy of the neural network detector.

#### 3.3.1. Fast Gradient Sign Method (FGSM)

The FGSM (Fast Gradient Sign Method) is a technique used to generate adversarial examples. It adds a small perturbation to the input image, causing the neural network to misclassify the image. This perturbation is computed in the direction of the gradient of the loss function with respect to the input image, aiming to maximize the loss function and deceive the model. The specific formulation is given by Equation (10):

(10) $$x^{\prime }=x+\epsilon \cdot \mathrm{sign}(\nabla_{x}J(\theta ,x,y))$$

where $x^{\prime }$ is the adversarial example, ϵ represents the size of the perturbation, and sign denotes the gradient of the loss function $\nabla_{x}J(\theta ,x,y)$ with respect to the input image x.

#### 3.3.2. Projected Gradient Descent (PGD)

PGD is an iterative extension of the FGSM, which optimizes the adversarial example through multiple iterations, making the generated adversarial sample more potent. Its principle is given by Equation (11):

(11) $$x_{t+1}^{\prime }={\mathrm{Proj}}_{x,\epsilon }\left(x_{t}^{\prime }+\alpha \cdot \mathrm{sign}(\nabla_{x}J(\theta ,x_{t}^{\prime },y))\right)$$

The step size for each iteration, denoted by α, controls the magnitude of the update, while the projection operation, represented by ${\mathrm{Proj}}_{x,\epsilon }$, governs the range of the sample projection. After undergoing adversarial training, the adversarial stego-image, formed by adversarial attacks, reduces the accuracy of the neural network [33,34] detector, causing misclassification and ultimately protecting the secret image.

### 3.4. Improving the Quality of Adversarial Stego-Images Using Generative Adversarial Networks

Generative adversarial networks (GANs) typically consist of a generator and a discriminator, which are trained together through an adversarial process. The goal of the generator is to produce increasingly realistic samples, while the discriminator works to distinguish whether these samples are real or fake. As training progresses, the generator becomes more adept at creating samples that closely resemble real data, while the discriminator continuously improves its ability to identify them. In the standard GAN, real samples are represented as x, latent vectors as z, and the loss function is expressed as Equation (12).

(12) $$\underset{G}{min}\underset{D}{max}\;V(D,G)=E_{x∼p_{data}(x)}[\log D(x)]+E_{z∼p_{z}(z)}[\log(1-D(G(z)))]$$

In this paper, we replace the standard generator with the encoder, using the stego-image ($C^{\prime }$) generated by the encoder as the output of the generator. The encoder and discriminator together form a GAN [35]. In this way, the discriminator learns to distinguish the differences between $C^{\prime }$ and ${C^{\prime }}_{adv}$, and it feeds back the discrimination results to the encoder, further improving the quality of the stego-image generated by the encoder.

Covertness of Perturbations: In traditional steganographic methods, the generated stego-images may reveal the hidden secret information. However, perturbations generated by GANs, after adversarial training, can make the stego-images visually similar to the original images, with the perturbation part being difficult to detect. Specifically, during each update of the generator, it optimizes the perturbations to make them resemble real images while enhancing their visual and structural covertness. The design goal of these perturbations is to make the stego-images nearly indistinguishable from real images in appearance, thereby reducing the likelihood of detecting stego-images using traditional image analysis techniques.

Adversarial Perturbations Enhance Stego-Image Security: In the adversarial training of GANs, the generator not only creates realistic stego-images but also applies perturbations to these images, making them more complex and resistant to analysis. These perturbations are continuously optimized through the adversarial process, effectively countering steganalysis (discriminator) models. Even if the stego-image undergoes further classification or analysis, extracting the hidden information becomes significantly more challenging. Therefore, the presence of perturbations makes it more difficult for attackers to extract hidden information using steganalysis techniques (e.g., deep learning models).

Adaptiveness of Perturbations Enhances Deceptiveness Against Steganalysis: During each adversarial training cycle, the generator adjusts its perturbation strategy based on feedback from the discriminator, making the generated stego-images more difficult to identify as fake. The discriminator learns to distinguish between real and forged stego-images, while the generator continually fine-tunes the perturbations after each training session, gradually producing stego-images that are both more covert and more resilient to adversarial detection. Due to this adaptive adjustment capability, the generator can continuously optimize the quality of the perturbations, further enhancing the security of the stego-images.

Through these perturbations, GAN-generated stego-images are not only visually covert but also enhance resistance to steganalysis attacks. Attackers are confronted with complex and adversarial perturbations, which significantly increase the difficulty of extracting secret information. As a result, through adversarial training, the generator can produce more secure stego-images, thus improving the security and effectiveness of steganographic techniques in practical applications.

### 3.5. The Three Stages of the AGASI Framework

#### 3.5.1. Stego-Image Generation and Extraction Stage

**Figure 3:**
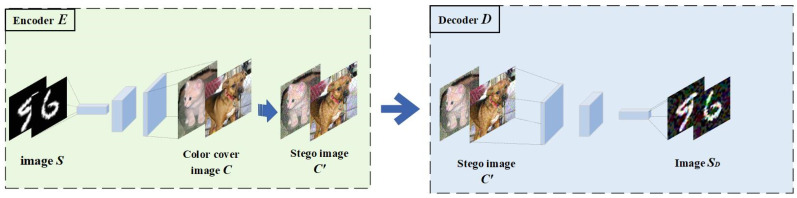
The embedding and extraction of the secret grayscale image S, where $S_{D}$ represents the secret information extracted by the decoder without any added adversarial perturbations.

The encoder and decoder play crucial roles in the entire process. The encoder combines the secret image with the cover image using a specific algorithm to generate the stego-image, ensuring that it remains visually indistinguishable from a regular image and thereby concealing the secret information. On the other hand, the decoder employs a specific decryption method to extract the original secret image from the stego-image, ensuring secure transmission and accurate recovery of the information. The process of generating and extracting the stego-image is illustrated in Figure 3.

In Figure 3, the grayscale image (S) is the secret image to be transmitted, which is input to the encoder along with the color cover image (C). Then, based on Equation (9), the losses for the encoder and decoder are calculated and the weights are updated. Eventually, the encoder (E) generates the stego-image, and the decoder (D) extracts the secret image ($S_{D}$).

The generation of stego-images is accomplished by both the encoder and the generator. The encoder embeds the secret information into the cover image to generate the stego-image, while the generator further optimizes the image’s covertness and adversarial robustness through adversarial training. The role of the encoder is to embed the secret image (S) into the cover image (C), resulting in the stego-image ($C^{\prime }$). This process is implemented through a multi-layer convolutional network, ensuring that the generated stego-image not only hides the secret information but also minimizes the visual difference from the original cover image.

Firstly, the first layer of the encoder (ConvolutionBlock1) performs initial feature extraction through a 3 × 3 convolution, batch normalization (BN), and the LeakyReLU activation function. The primary task of this layer is to help the network identify low-level features in the cover image, such as edges, textures, and simple shapes. The 3 × 3 convolution operation captures features from local regions, while batch normalization helps accelerate the training process and improves the stability of the network. The LeakyReLU activation function addresses the “dead neuron” issue that can occur with ReLU, while maintaining some activation in the negative value region to prevent information loss.

The second to eighth layers of the encoder employ Inception modules for multi-scale feature extraction. A key advantage of the Inception module is its ability to extract information at various scales by parallelizing multiple convolution branches, allowing it to adapt to features of differing sizes and complexities within the image. Each branch includes convolution kernels or pooling operations of varying sizes, enabling it to capture features at different levels and scales from the cover image. Specifically, Branch 1 utilizes a 1 × 1 convolution to compress features, thereby enhancing the network’s non-linear expressiveness, followed by batch normalization (BN) and ReLU activation. A subsequent 3 × 3 convolution extracts more complex local features. Branch 2 also begins with a 1 × 1 convolution, followed by BN and ReLU activation, and then uses a larger 5 × 5 convolution kernel to capture features from a broader range in the cover image. Branch 3 mirrors Branch 2 but uses an additional set of 5 × 5 convolutions to further capture multi-scale information. Branch 4, in contrast, employs a 3 × 3 max pooling operation to extract primary structural features over a larger area of the image. A 1 × 1 convolution, followed by BN and ReLU activation, is applied to the pooled features to further enhance the image’s expressiveness.

Under the influence of these branches, the encoder can extract rich feature information from the cover image at different scales and orientations, progressively capturing the complexity and details of the image, thereby enhancing the quality and concealability of the stego-image. Subsequently, the ninth layer (ConvolutionBlock1) integrates and refines the previously extracted features. By applying convolution operations, batch normalization (BN), and LeakyReLU activation functions once again, this layer further strengthens the high-level features in the image, thereby generating high-quality stego-images.

Finally, the output layer of the encoder uses a 1 × 1 convolution and a Tanh activation function to generate the stego-image ($C^{\prime }$). The 1 × 1 convolution compresses the feature map and ultimately generates the stego-image, while the Tanh activation function ensures that the pixel values of the image remain within a reasonable range. The stego-image ($C^{\prime }$) is visually indistinguishable from the original cover image (C), and yet it contains the hidden information of the secret image. In this way, the encoder not only successfully embeds the secret image into the cover image but also ensures the concealability and quality of the stego-image, making it difficult for the hidden information to be detected.

After generating the stego-image, the total loss of the encoder and decoder is calculated according to formula (9). This process involves computing the difference between the stego-image ($C^{\prime }$) and the cover image (C), as well as the error between the decoder’s recovered secret image ($S^{\prime }$) and the true secret image (S). The loss function consists of these two components, assessing both the quality of the stego-image and the recovery performance of the secret information. Then, using the backpropagation algorithm, the weights of the encoder and decoder are updated based on the computed loss values. This process is performed during each training iteration, gradually optimizing the model’s performance, and thereby improving the quality and concealability of the stego-image and ensuring the accurate recovery of the secret information. The process is represented in Algorithm 1.
**Algorithm 1:** Embedding and extraction of secret information.**Input:**Color cover image C, secret image S
**Output:**Stege image $C^{\prime }$, secret image $S_{D}$
1:   Initialize Encoder Parameters θ, Decoder Parameters ∅
2:***for*** i ←1 to e ***do***3:   C← Input Color Image 4:   S← Input Secret Image (Gray) 5: //Encoder
6:   
$C^{\prime }=Encoder(\theta^{\left(i-1\right)},C,S)$
7: //Encoder Structure:8:   Layer 1: ConvolutionBlock1 (3 × 3 Conv + BN + LeakyReLU)9:   Layers 2–8: Inception Modules with parallel branches (1 × 1 Conv + BN + ReLU, 3 × 3 Conv, 5 × 5 Conv, MaxPooling + Conv)10:   Layer 9: ConvolutionBlock1 (3 × 3 Conv + BN + LeakyReLU)11: Output: ConvolutionBlock2 (1 × 1 Conv + Tanh) to generate Stego-image $C^{\prime }$
12:   
$SD=Decoder(\emptyset^{\left(i-1\right)},C^{\prime })$
13: //Decoder Structure:14:   Layers 1–5: ConvolutionBlock1 (3 × 3 Conv + BN + LeakyReLU)15: Output: ConvolutionBlock2 (1 × 1 Conv + Tanh) to extract Secret Image $S_{D}$
16:   
$Loss_{E}^{i}=Loss_{E}(Encoder(\theta \left(i-1\right),C,S))$
17:   
$Loss_{D}^{i}=Loss_{D}(SD,S)$
18:   Backpropagate $\left(\lambda (E)\;*\;Loss(E^{i})\;+\lambda (D)\;*\;Loss(D^{i})\right)$
19: Update Encoder Parameters $\theta^{i}$, Decoder Parameters $\emptyset^{i}$
20:***End for***21:   
$\theta =\theta^{e},\emptyset =\emptyset^{e}$


#### 3.5.2. Adversarial Stego-Image Generation Phase

**Figure 4:**
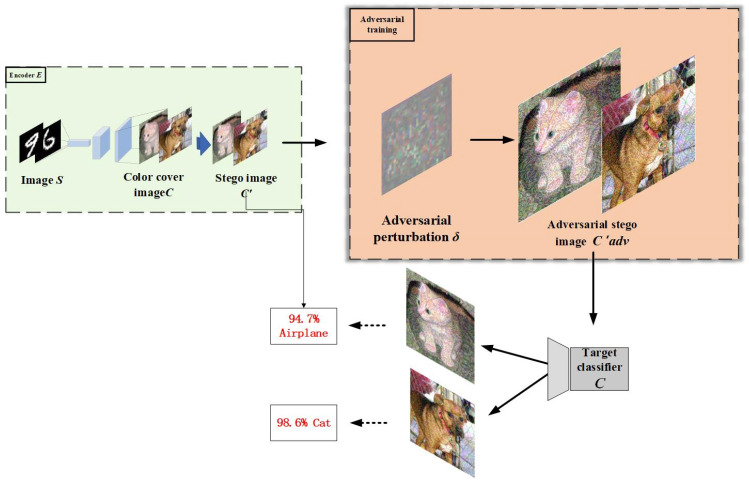
In this process, the stego-image undergoes adversarial attacks to generate an adversarial stego-image (${C^{\prime }}_{adv}$), which deceives the neural network analyzer, causing misclassification and enhancing the security of the stego-image.

In steganography, a stego-image is typically generated by embedding the secret image into the cover image. However, directly using the stego-image may easily be detected by trained neural networks or other analysis tools as $C^{\prime }$, thereby exposing the features of the secret image. Therefore, to enhance the security of the stego-image, adversarial attacks are applied to generate a stego-image with adversarial perturbations, which deceive the neural network analyzer, reducing the classification accuracy of the network and making it difficult for third parties to extract the secret image information through analysis. The goal of the adversarial stego-image (${C^{\prime }}_{adv}$) is to reduce the accuracy of the neural network, leading to misclassification, and thus preventing third parties from extracting the secret image features through neural network analysis, thereby protecting its security. The generation process is shown in Figure 4.

By applying adversarial attacks to the stego-image, adversarial stego-images (${C^{\prime }}_{adv}$) are generated. These attacks apply perturbations computed and imposed using the Fast Gradient Sign Method (FGSM) and Projected Gradient Descent (PGD). Specifically, the FGSM and PGD calculate the gradient of the loss function of the image and apply perturbations based on this calculation to optimize the stego-image, decreasing its classification accuracy and “deceiving” the neural network analyzer, resulting in misclassification. This process iteratively optimizes the size and direction of the perturbation, updating and fine-tuning it with each iteration based on the current classification error of the image, further enhancing its adversarial robustness.

This not only makes the generated adversarial stego-image (${C^{\prime }}_{adv}$) more challenging to classify correctly but also preserves a good visual quality, minimizing any loss in image quality. As training progresses, the difficulty for the neural network analyzer in processing these adversarial stego-images increases, eventually rendering it impossible to accurately extract the hidden secret information. Therefore, the final adversarial stego-image (${C^{\prime }}_{adv}$) not only improves concealability but also strengthens the security of the image, effectively preventing third parties from extracting secret information through image analysis.

In Figure 4, the generated adversarial stego-image (${C^{\prime }}_{adv}$) is first input into a target classifier, which attempts to classify the image into one of several predefined categories. Due to the adversarial perturbations introduced during the generation process, the stego-image is more likely to be misclassified, being assigned to an incorrect category with higher probability. This misclassification is intentional, as it strengthens the adversarial nature of the image, making it more difficult for any classifier or deep learning model to accurately recognize the hidden secret information.

The primary advantage of this process is that even if a third party, equipped with a pre-trained deep learning model hosted in the cloud, attempts to retrieve the hidden information embedded within the image, they will face significant challenges. The adversarial perturbations hinder the model from accurately identifying and extracting the secret content, ensuring the preservation of the image’s privacy. This approach effectively protects the stego-image from detection or decryption attempts, adding an additional layer of security during the transmission of secret images. The process is illustrated in Algorithm 2.

However, this approach often results in a significant decrease in image quality while enhancing adversarial robustness, which can be inconvenient for users. Therefore, although the stego-image shows a substantial improvement in privacy protection, its quality may be compromised, leading to some visual distortion. To balance the trade-off between adversarial robustness and image quality, we introduce generative adversarial networks (GANs). By leveraging GAN training, we can improve the adversarial robustness of the stego-image while preserving its high visual quality, thus avoiding the image quality degradation commonly associated with traditional methods.
**Algorithm 2:** Generate Adversarial Stego-images ${C^{\prime }}_{adv}$
**Input:**Stego-image $C^{\prime }$‘, Encoder’s Parameters θ, Neural Network’s Parameters W, Attack Type (*FGSM*/*PGD*), Perturbation Magnitude ε, Number of Iterations T(for *PGD*), Step Size α(for *PGD*)**Output:**Adversarial Stego-images ${C^{\prime }}_{adv}$
1:***If*** Attack Type == *FGSM*
***then***2:   //FGSM Attack3:   
$\nabla (C^{\prime })=\nabla (C^{\prime }\;Loss(C^{\prime },W))$
4:   Generate Perturbation:5:   
$\delta \_FGSM=\varepsilon \;*\;sign(\nabla \_C^{\prime })$
6:   Apply Perturbation:7:    
${C^{\prime }}_{adv}=C^{\prime }+\delta \_FGSM$
8:***else if*** Attack Type == PGD ***then***
9:   //*PGD* Attack10:   Initialize Perturbation δ=0
11: ***for*** t ← 1 to T ***do***12:   
$\nabla (C^{\prime })=\nabla (C^{\prime }\;Loss(C^{\prime },W))$
13:   Update Perturbation:14:   
$\delta^{t}=\delta^{\left(t-1\right)}+\alpha \;*\;sign(\nabla (C^{\prime }))$
15:   Clip Perturbation:16:   
$\delta^{t}=Clip\left(\delta^{t},-\varepsilon ,\;\varepsilon \right)$
17:***end for***18:   Apply Perturbation:19:   
${C^{\prime }}_{adv}=C^{\prime }+\delta^{T}$
20:***end if***21:**return** ${C^{\prime }}_{adv}$

#### 3.5.3. Training Phase of the Generative Adversarial Network

**Figure 5:**
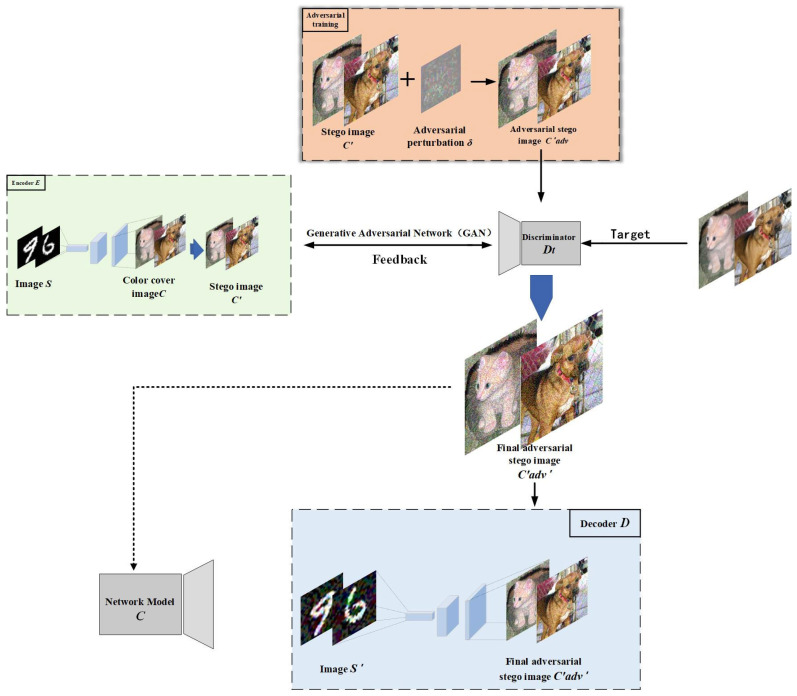
The encoder replaces the generator. Together with the discriminator, they form a GAN. The generated ${C^{\prime }}_{adv^{\prime }}$ makes it difficult for third parties to accurately identify the secret information in the image through deep learning models, while the secret information (S) extracted by the decoder maintains high quality.

During the training phase of the generative adversarial network (GAN), the encoder functions as the generator and, together with the discriminator, forms the complete GAN architecture. In this context, the discriminator is specifically designed as a steganalyzer that differentiates between real images and stego-images, while the generator’s objective is to produce stego-images through adversarial learning. The loss function of the GAN is calculated using Formulas (7) and (13), which capture the adversarial dynamics between the generator and the discriminator. By continuously optimizing and updating the weights, the network gradually generates stego-images that closely resemble the real secret image and ultimately outputs an image that is highly similar to the original secret image. The primary goal of the network is to enhance the correlation between C and ${C^{\prime }}_{adv}$, ensure the effectiveness of the adversarial perturbations, and improve the quality of secret information (S) extraction, ensuring that the steganography process is not only highly concealed but also allows accurate recovery of the secret information during decoding. The entire process is shown in Figure 5.

In this phase, the core objective of the generative adversarial network (GAN) is to continually optimize the quality of the generated adversarial steganographic image (${C^{\prime }}_{adv}$) through the iterative competition between the generator (encoder) and the discriminator (steganalyzer). The generator (encoder) and the steganalyzer (discriminator) are jointly optimized to enhance both the realism of the stego-images and their ability to deceive the steganalyzer. The generator (encoder) is tasked with creating the steganographic image ($C^{\prime }$) from the color image (C) and the secret image (S), while the steganalyzer (discriminator) learns to differentiate between real steganographic images and adversarial steganographic images during training. In the training process, the generator (encoder) first generates the steganographic image ($C^{\prime }$), which contains the information of the secret image (S). Then, the steganographic image ($C^{\prime }$) undergoes an adversarial attack (FGSM or PGD) to generate the adversarial steganographic image (${C^{\prime }}_{adv}$). This image visually resembles ($C^{\prime }$) but contains perturbations designed to mislead the steganalyzer, reducing its classification accuracy and enhancing the security of the stego-image.

The generated adversarial steganographic image (${C^{\prime }}_{adv}$) is input into the steganalyzer (discriminator) for training. The goal of the discriminator is to determine whether the image is a real steganographic image. During this process, the discriminator constantly adjusts its parameters and learns to distinguish between (C) and (${C^{\prime }}_{adv}$). Meanwhile, the goal of the generator (encoder) is to optimize its generation strategy so that (${C^{\prime }}_{adv}$) becomes increasingly difficult for the discriminator to classify as a “fake” image. This dynamic process of generating and analyzing stego-images leads to a constant feedback loop, where the generator and steganalyzer continuously refine their strategies based on each other’s performance.

Through adversarial training, the generator continuously improves, producing more realistic steganographic images while maintaining their adversarial characteristics to enhance security. The core of this process lies in the competition between the generator and the discriminator. The generator adjusts its generation strategy based on feedback from the discriminator during each iteration, thereby refining the quality of the steganographic image. The discriminator (Dt) is trained by maximizing the probability of correctly identifying color image (C) and minimizing the error in identifying adversarial steganographic images (${C^{\prime }}_{adv}$). On the other hand, the generator (E) is trained by minimizing the discriminator’s classification error of (${C^{\prime }}_{adv}$), continually improving the quality of the adversarial steganographic images (${C^{\prime }}_{adv}$) to make them more realistic. Through continuous training, the generator can eventually generate higher-quality steganographic images (${C^{\prime }}_{adv^{\prime }}$) that not only possess adversarial properties, reducing the accuracy of the neural network classifier, but also allow for the precise extraction of the secret image ($S^{\prime }$) during decoding. The process is depicted in Algorithm 3.
**Algorithm 3:** Training of GAN and Extraction of Secret Image $S^{\prime }$**Input:**Color Image C, Secret Image S, Decoder Parameters ∅, Discriminator Parameters D
**Output:**Final Generated Adversarial Stego-image ${C^{\prime }}_{adv^{\prime }}$, secret image $S^{\prime }$
1:***for*** each training iteration ***do***2:  
$C^{\prime }=E\left(\theta (C,\;S)\right)$
3:  ***if*** FGSM ***then***4:    
$C^{\prime }adv=FGSM\left(C^{\prime },\;\varepsilon \right)$
5:  
***else if*** 
PGD 
***then***
6:    
$C^{\prime }adv=PGD\left(C^{\prime },\;\varepsilon ,\;\alpha \right)$
7:  
***end if***
8:    
$D(real)=D\left(C\right)$
9:    
$D(fake)=D\left({C^{\prime }}_{adv^{\prime }}\right)$

    //Compute Discriminator Loss L_D using Formula (13)10:    
$L(D)=-log\left(D(real)\right)-log\left(1-D(fake)\right)$

    //Compute Generator Loss L_G using Formula (7)11:    
$L\_G=-log\left(D(fake)\right)$
12    
$S^{\prime }=Decoder\_\emptyset \left(C^{\prime }adv^{\prime }\right)$
13:    
$L(E)=Loss\left(S^{\prime },\;S(true)\right)$
14:    Backpropagate L(E) and update Decoder’s parameters ∅
15:  
***end for***
16:**return** ${C^{\prime }}_{adv^{\prime }}$, $S^{\prime }$

## 4. Experiments and Results

All experiments were conducted in the PyCharm development environment, utilizing the PyTorch framework to build and train deep learning models. The hardware used in the experiments included a workstation equipped with an NVIDIA GPU, which accelerated the model training process. The datasets used for the experiments were the MNIST, CIFAR-10, and LFW datasets. Specifically, MNIST grayscale images were embedded into CIFAR-10 color images, while LFW grayscale images were embedded into their corresponding color images. Based on the images generated at different stages, the adversarial and embedding performance of the AGASI method was evaluated on several classic neural network classification models, including ResNet50, VGG16, Wide ResNet, DenseNet121, and DenseNet-40.

### 4.1. Performance of CIFAR-10 and MNIST Datasets for Each Image

In this section, we embed MNIST grayscale images into CIFAR-10 color images. Different images at various stages are generated through the three phases mentioned above, and a comparative analysis is conducted. Among these generated images, the most important are the ${C^{\prime }}_{adv}$, ${C^{\prime }}_{adv^{\prime }}$, and $S^{\prime }$ images, as they directly relate to the performance of steganography. To evaluate the performance of these images, we designed and conducted the following experiments.

#### 4.1.1. The Attack Success Rate of ${C^{\prime }}_{adv}$

The attack success rate refers to the probability or percentage of adversarial samples that successfully “deceive” the classifier. It measures the proportion of instances in which the model misclassifies adversarial examples generated by adding perturbations. A successful attack occurs when the predicted class of a perturbed image does not match its true class.

**Figure 6:**
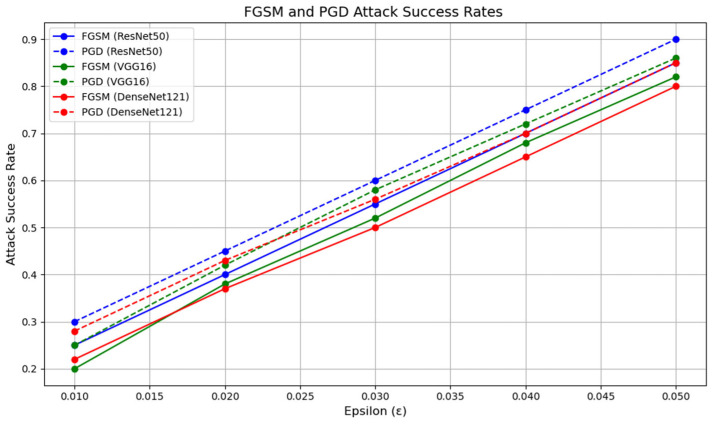
Success rate curves of different neural network models under FGSM and PGD attacks. The horizontal axis represents the perturbation strength, while the vertical axis shows the attack success rate. The blue line corresponds to the ResNet50 model, the green line to the VGG16 model, and the red line to the DenseNet121 model. Solid lines indicate the success rate under the FGSM attack, while dashed lines represent the success rate under the PGD attack. These curves offer a clear comparison of the degrees of robustness of different models across varying attack strengths.

For the performance evaluation of ${C^{\prime }}_{adv}$, we first embedded MNIST grayscale images into CIFAR-10 images. We selected three classic deep learning models for testing: ResNet50, a deep residual network known for alleviating the vanishing gradient problem; VGG16, a well-established convolutional neural network recognized for its deep architecture and simple convolutional blocks; and DenseNet121, a densely connected network that enhances feature reuse by connecting all layers. We trained each model for 100 epochs on the CIFAR-10 dataset and generated adversarial samples using the FGSM and PGD attack methods. The perturbation strength (ε) was varied within the range of 0.01 to 0.05, and the attack success rates were evaluated for different perturbation strengths. The impact of perturbation strength (ε) on the attack success rate is shown in Figure 6.

From Figure 6, it can be observed that as the perturbation strength (ε) increases, the attack success rate rises significantly. This suggests that as the perturbation becomes larger, the effect of adversarial examples on the model becomes more pronounced, leading to a higher attack success rate. For ResNet50 and DenseNet, relatively high attack success rates can be achieved even at smaller perturbation strengths (e.g., ε = 0.01), indicating that these two models are more vulnerable to small perturbations. Notably, ResNet50 reaches a high attack success rate at ε = 0.01, with its attack success rate increasing rapidly as the perturbation strength grows. In contrast, VGG16 demonstrates stronger robustness. At smaller perturbation strengths (e.g., ε = 0.01), the attack success rate of VGG16 is significantly lower than that of the other two models, indicating that VGG16 is more stable in the presence of perturbations. However, as the perturbation strength increases, the attack success rate of VGG16 gradually rises. When the perturbation strength (ε) reaches 0.04, the attack success rates of all models approach 100%, indicating that even a more robust model like VGG16 cannot withstand adversarial attacks at higher perturbation levels.

**Figure 7:**
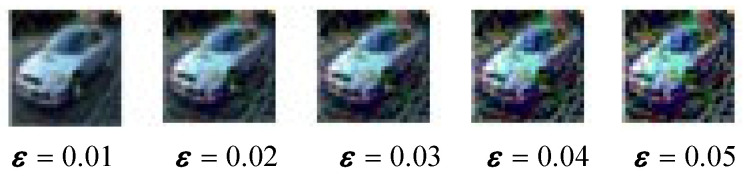
As the perturbation strength increases, the quality of the steganographic image (${C^{\prime }}_{adv}$) deteriorates.

However, this raises an issue: although the attack success rate increases significantly at higher perturbation strengths, the image quality of the adversarial examples deteriorates noticeably. For instance, when using the PGD attack with ε = 0.005, the quality of the generated image becomes very poor, as shown in Figure 7.

#### 4.1.2. Visual Effect of the Image

In the above experiment, ResNet50 outperformed VGG16 and DenseNet121. To further assess the impact of adversarial perturbations on image visual quality, we evaluated it using three metrics: Peak Signal-to-Noise Ratio (PSNR), Structural Similarity Index (SSIM), and Structural Mismatch Error (SME). PSNR measures the signal-to-noise ratio of an image, indicating its overall quality. SSIM evaluates the structural similarity of the image, considering factors such as brightness, contrast, and structural information, and provides a visual quality assessment. SME, on the other hand, assesses structural mismatches after image reconstruction or processing, quantifying the loss or alteration of structural details. By combining these three metrics, we can comprehensively evaluate image quality from multiple perspectives.

The quality of the stego-image ($C^{\prime }$) and the adversarial stego-image (${C^{\prime }}_{adv}$) directly impacts the effectiveness of secret information concealment. In this experiment, FGSM and PGD attacks were applied, and the quality levels of both the stego-image ($C^{\prime }$) and adversarial stego-image (${C^{\prime }}_{adv}$) were assessed under varying perturbation conditions. The results are presented in Table 1.

The first row of data in Table 1 shows the PSNR, SSIM, and SME values of the cover image and steganographic image without any perturbation. In the absence of any attack, the PSNR value reaches 32.924, indicating good visual quality and reflecting the high encoding performance of the encoder. The other data points represent the PSNR, SSIM, and SME values of the cover image and adversarial steganographic image under different perturbation intensities of FGSM and PGD attacks, which provide insights into the quality of the adversarial steganographic image. As can be observed, with an increase in perturbation intensity, both PSNR and SSIM values decrease, while the SME value increases. Additionally, FGSM attack demonstrates a better performance at lower perturbation intensities, while PGD attack outperforms the FGSM as the perturbation intensity increases. As the perturbation intensity increases, the PSNR values of both the steganographic image and the adversarial steganographic image significantly decrease, indicating a noticeable decline in image quality under severe perturbations, which affects the protection of the secret image. Therefore, to improve image quality and enhance the steganographic performance, we consider employing the GAN to address this issue.

#### 4.1.3. The Performance of the Generative Adversarial Network (GAN)

The introduction of the GAN improves the quality of adversarial steganographic images while effectively reducing the accuracy of the neural network classifier. This allows the image quality to be maintained while diminishing the classifier’s performance at higher perturbation intensities. The final quality results of the generated adversarial steganographic image (${C^{\prime }}_{adv^{\prime }}$) are shown in Table 2.

**Figure 8:**
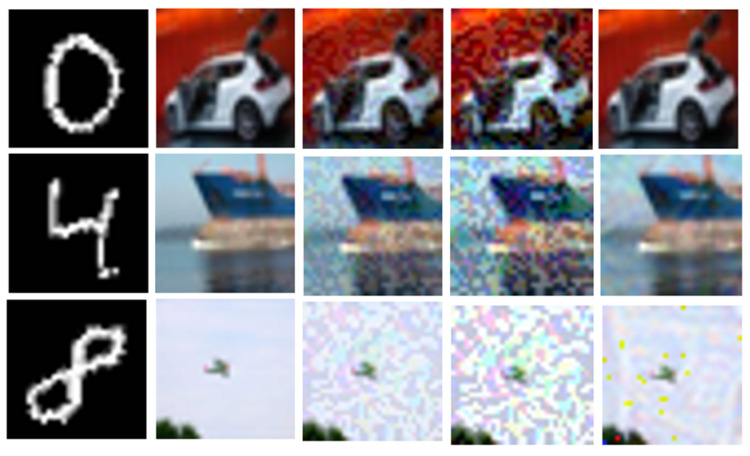
The first column represents the secret image to be hidden (S); the second column is the color cover image (C); the third column is the steganographic image generated by the encoder ($C^{\prime }$); the fourth column shows the adversarial steganographic images generated at PGD attack perturbation strengths of 0.01, 0.03, and 0.05, from top to bottom (${C^{\prime }}_{adv}$); the last column shows the final adversarial steganographic image generated after processing through the GAN with a PGD perturbation strength of 0.10 (${C^{\prime }}_{adv^{\prime }}$). It can be observed that the GAN effectively improves the quality of the images formed after adversarial attacks.

The data in Table 2 are compared with that in Table 1. When the FGSM perturbation strength is 0.04, the PSNR value of the adversarial steganographic image (${C^{\prime }}_{adv^{\prime }}$) generated using the GAN is 29.863, while the PSNR value of the adversarial steganographic image without the GAN is 24.451. As the attack strength increases, the PSNR value with the GAN at a perturbation strength of 0.10 is still slightly higher than the PSNR value of 0.03 without the GAN. Additionally, the performance in SSIM and SME also shows excellent results. The performance under PGD attack is similar, with the attack performance being better than that of FGSM. To better observe the visual effect of the generative adversarial network (GAN) on images, the PGD method, which performs well in attacks, was used to perturb the images. The final generated images after PGD attack are shown in Figure 8.

After adjustment by the GAN, the quality of the images has been improved. However, to ensure the effectiveness of the steganographic method, it is also necessary to evaluate its impact on the neural network classification performance, especially its attack success rate. To further investigate the effect of perturbation on the attack success rate, we selected 100 images from each of the ten categories of CIFAR-10 for processing and generation ${C^{\prime }}_{adv^{\prime }}$. At a perturbation strength of 0.10, we used the ResNet50 model to analyze the generated sparse matrix and plotted the heatmap. The results are shown in the figure below.

**Figure 9:**
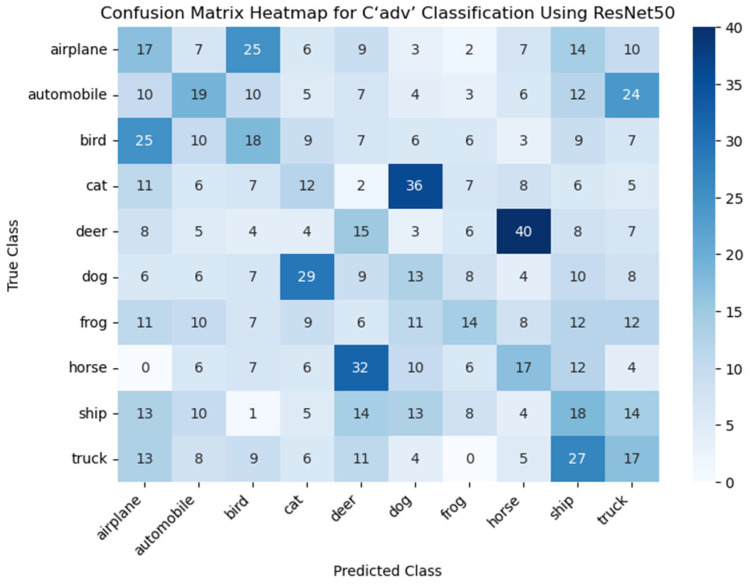
The confusion matrix heatmap is used to evaluate the classification performance of the ResNet50 model on ${C^{\prime }}_{adv^{\prime }}$. The confusion matrix is a tool for assessing the performance of a classification model, showing the relationship between the predicted and true labels of the model for each category. The *X*-axis represents the predicted classes (Predicted Class), which are the output predictions of the ResNet50 model for the images, while the *Y*-axis represents the true classes (True Class), which are the actual labels of the images. The value in each cell indicates the number of images that belong to the true class but are predicted as a specific class. Darker colors indicate a higher number of predictions for that category, while lighter colors suggest fewer predictions.

In Figure 9, it can be observed that the classification counts for certain categories are significantly higher than others. For example, images of cats are misclassified as dogs, and images of deer are misclassified as horses. We attribute this to the fact that these two categories share similar features. The diagonal in the heatmap represents the number of images from ${C^{\prime }}_{adv^{\prime }}$ that were correctly classified by ResNet50 without error. From the figure, it can be concluded that, at a disturbance strength of 0.10, the attack success rate for ${C^{\prime }}_{adv^{\prime }}$ is 84%. At the same disturbance strength, ${C^{\prime }}_{adv}$ achieves nearly 100% classification accuracy. However, it is worth noting that at this point, the PSNR value for ${C^{\prime }}_{adv}$ is noticeably lower than for ${C^{\prime }}_{adv^{\prime }}$. Additionally, when comparing both categories with the same PSNR value, the attack success rate for ${C^{\prime }}_{adv^{\prime }}$ is significantly higher than that of ${C^{\prime }}_{adv}$, with an increase of approximately 23.31%. This suggests that by introducing the GAN, the increasing disturbance strength not only enhances the adversarial robustness of the image but also effectively improves its quality. Overall, the presence of the GAN successfully maintains the adversarial nature of the image while improving its visual quality.

#### 4.1.4. Performance of Secret Information

The extraction of secret information is directly related to the steganographic capability of the steganographic technique. In this experiment, the extracted secret information is a grayscale secret image, so the quality of the extracted secret image is crucial. The adversarial attack used in the experiment is the PGD attack, which demonstrates a better performance. The experimental data are shown in the table below. Here, $S_{adv}$ represents the secret image extracted from the adversarial stego-image without the introduction of the GAN.

In Table 3, the PSNR value of the original encrypted image and the output from the decoder without any attack or perturbation is 30.932. After the introduction of perturbation attacks, the extracted secret image shows some distortion; however, this distortion remains within a controllable range. Overall, after incorporating the GAN, the quality of the secret image improves, as reflected in both the PSNR and SSIM metrics, compared to the case where the GAN is not used.

**Figure 10:**
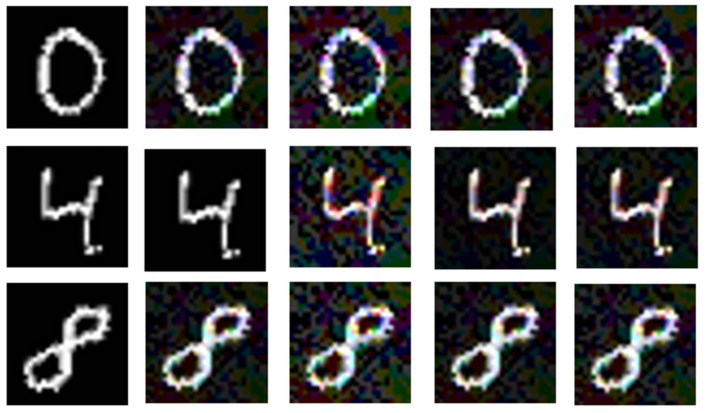
The results under PGD attack are shown. The first column displays the secret information (S). The second column shows the extracted secret information ($S_{D}$) from the decoder without any perturbation. The third column shows the extracted secret image ($S_{adv}$) from the adversarial stego-image with a perturbation strength of 0.04. The fourth column shows the extracted secret image ($S^{\prime }$) from the adversarial stego-image with a perturbation strength of 0.04. The last column displays the extracted secret image ($S^{\prime }$) from the adversarial stego-image with a perturbation strength of 0.10.

As shown in Figure 10, at a perturbation strength of 0.10, the quality of the extracted secret information is higher than that without the GAN. However, some distortion still occurs at this perturbation strength. As shown in Figure 6, the adversarial success rate at a perturbation strength of 0.05 is already quite high, so increasing the perturbation strength to 0.10 is unnecessary. This suggests that the GAN improves the quality of secret information extraction, especially under higher perturbation strengths, where the improvement is more pronounced.

### 4.2. Steganographic Analysis Capability of AGASI

In Section 4.1, we investigated the performance of the final generated adversarial stego-images within adversarial neural network classifiers, as well as their image quality in the context of multi-class classification tasks. Since steganalysis models are typically more suited to binary classification tasks rather than multi-class scenarios, this section focuses primarily on evaluating the steganalysis capability of AGASI in the context of binary classification.

To assess the steganalysis performance of AGASI, we applied it to the LFW dataset for face recognition tasks. LFW (Labeled Faces in the Wild) is a widely used benchmark dataset for face verification, designed to test a model’s accuracy in distinguishing between pairs of images labeled as “same person” or “different person”. Through this transfer learning experiment, we not only validated the generalization capability of the AGASI model when handling diverse datasets but also examined its ability to maintain a robust performance in larger and more challenging tasks. To achieve this, we compared the performance of the AGASI model with two baseline models, Wide ResNet and DenseNet-40, in the face verification task.

#### 4.2.1. Performance of Anti-Steganalysis Ability

**Figure 11:**
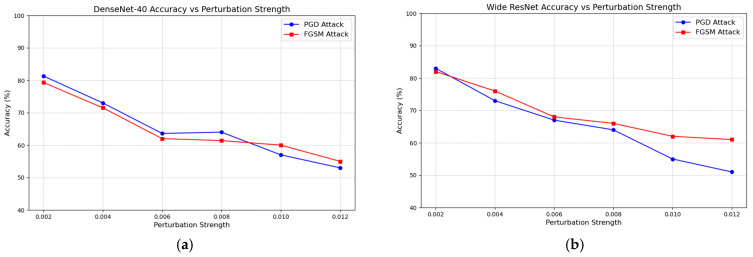
(**a**,**b**) shows Variations in steganalysis performance of the DenseNet-40 and Wide ResNet models under different perturbation strengths. The *x*-axis represents the perturbation strength of FGSM and PGD attacks, ranging from 0.002 to 0.012. The blue line represents the PGD attack, while the red line represents the FGSM attack. The *y*-axis represents the steganalysis performance of the neural network, which indicates the probability that it correctly classifies the images.

The experiment randomly selected 1000 stego-images and tested the impact of FGSM and PGD attacks under different perturbation conditions on the DenseNet-40 [24] and Wide ResNet [25] steganalysis models. The performance of the two models in the steganalysis task was evaluated. The experiment aims to observe the impact of adversarial perturbations on the accuracy of the models in identifying stego-images. The experimental results are shown in Figure 11.

In Figure 11, as the adversarial perturbation increases, both FGSM and PGD attacks lead to a significant decrease in the accuracy of the steganalysis model. At lower perturbation strengths, the effects of both attacks on the model’s steganalysis performance are similar. However, as the perturbation strength increases, particularly when it exceeds 0.008, PGD attacks show a superior performance, and the effect becomes more pronounced. This trend suggests that PGD attacks, with higher perturbation strengths, can significantly worsen the decline in model performance. Additionally, the performance difference of the Wide ResNet model under FGSM and PGD attacks is particularly noticeable, highlighting the varying effectiveness of these two adversarial attack strategies in the steganalysis task. This difference can be quantified and compared using the specific data in Table 4, providing a clearer picture of the performance variations under different attack conditions.

To more intuitively explore the impact on the steganalysis model, we created an AUC (Area Under the Curve) graph for FGSM and PGD attacks on Wide ResNet. The AUC graph illustrates the changes in the model’s performance in detecting stego-images at different attack strengths, reflecting the impact of adversarial examples on the model’s steganalysis capability.

**Figure 12:**
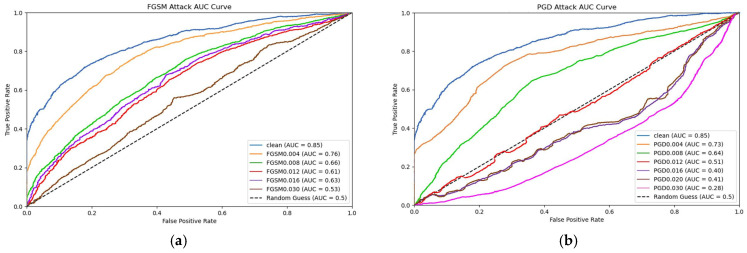
In figures (**a**,**b**), the horizontal axis represents the False Positive Rate (FPR), while the vertical axis represents the True Positive Rate (TPR). The area in the bottom-right corner of the curve in the graph corresponds to the AUC value, which indicates the detection accuracy of the Wide ResNet classifier.

To plot the AUC graph, we first used 1000 stego-image samples from the LFW dataset for steganalysis predictions. Then, by calculating the True Positive Rate (TPR) and False Positive Rate (FPR) for each sample, we obtained the ROC curve at different perturbation strengths and further calculated the AUC values. Finally, the AUC values at different perturbation strengths were plotted on the same graph to demonstrate the impact of adversarial perturbations on the performance of the Wide ResNet model in steganalysis. The generated AUC graph is shown in Figure 12.

As the perturbation strength increases, both FGSM and PGD attacks lead to a decline in the steganalysis performance of the Wide ResNet model. Notably, when the PGD perturbation exceeds 0.012, the Wide ResNet model is unable to differentiate between genuine and adversarial samples, with PGD outperforming FGSM in this regard. In the AUC graph generated by the PGD attack, further increases in perturbation strength result in a continuous decline in the model’s steganalysis performance. However, due to the presence of the GAN, the image quality remains preserved even as the attack strength increases.

#### 4.2.2. Effectiveness of LFW at Various Stages

**Figure 13:**
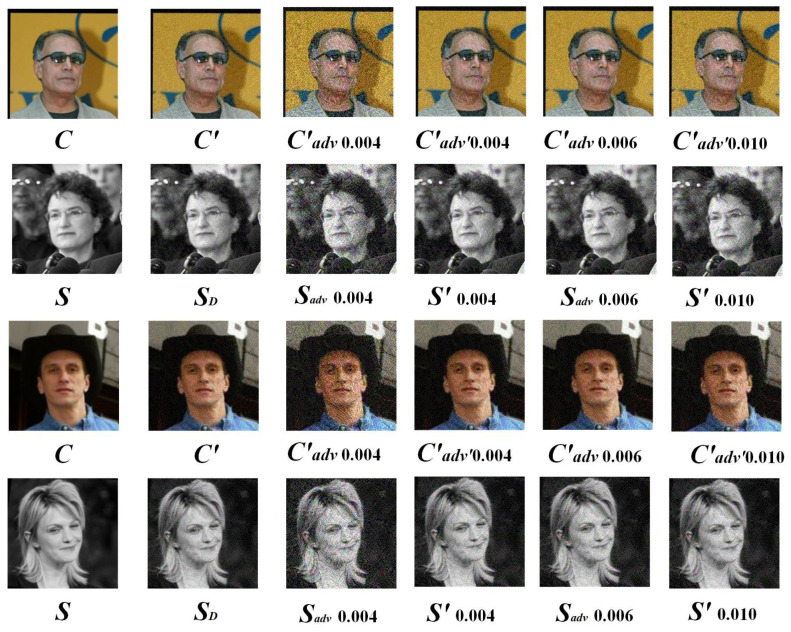
The first column displays the original images, the second column shows the images and their extractions generated in the first stage, the third column presents the images and their extractions generated in the second stage, and the fourth, fifth, and sixth columns illustrate the images and their extractions generated in the third stage.

Extracting secret information is a crucial step in steganography, directly influencing its performance. In this section, we compare the extracted secret information (S) from the LFW dataset with results from other extraction methods. We also analyze the PSNR and SSIM values of S under various perturbation intensities for three scenarios: $S_{D}$, $S_{adv}$, and $S^{\prime }$, to evaluate the robustness of the steganography. The relevant data can be found in Table 5. Furthermore, we compare the visual effects of images generated using AGASI under different perturbation conditions in the Wide ResNet model, with the specific results shown in Figure 13.

As shown in Figure 13, with an increase in perturbation strength, image distortion gradually intensifies, particularly under higher perturbation levels, where the image quality significantly deteriorates. However, the AGASI model continues to exhibit an excellent performance. For instance, in the images shown in the sixth column, when the perturbation strength is 0.010, both the generated and extracted images demonstrate a better quality than their counterparts in the third column. Moreover, AGASI shows strong adversarial robustness. At a PGD attack strength of 0.010, the Wide ResNet model can no longer distinguish whether the image is a stego-image, effectively enhancing the model’s steganographic capability while preserving its adversarial properties. These experiments reveal that AGASI not only excels on smaller datasets like CIFAR-10 but also performs well on larger pixel datasets such as LFW, showcasing its impressive transferability and robustness.

### 4.3. Embedding Capability of AGASI

Color images typically use a 24-bit color depth, while grayscale images utilize an 8-bit color depth, where each pixel is represented by an 8-bit grayscale value. Embedding a grayscale image within a color image involves inserting each pixel’s data from the grayscale image into one or more components of the color image’s pixels. As a result, the embedding capacity of the proposed method is 8 bits per pixel (bpp). In CIFAR-10, where the image size is 32 × 32, the embedding capacity of this method corresponds to 1 KB of secret information. Compared to other methods, this approach offers a larger embedding capacity. The specific embedding capacity is presented in Table 6.

## 5. Conclusions

This paper proposes AGASI, a GAN-based method for enhancing adversarial image steganography and addressing the issue of image quality degradation caused by increased perturbations. The method introduces perturbations into the stego-image by encoding the cover image and the secret grayscale image, effectively protecting the secret information. By leveraging the unique features of generative adversarial networks (GANs), this paper constructs an encoder and a discriminator, which work together to improve the quality of adversarial stego-images. The advantage of AGASI lies in its ability to not only disrupt the accuracy of the network model but also ensure the extraction of high-quality secret information. Experimental results show that, even under high perturbations, AGASI performs excellently in both image quality and adversarial robustness, with significant advantages over existing methods in terms of embedding capacity, effectively embedding secret information into the stego-image while maintaining visual quality. However, the computational efficiency of the method remains an important consideration, especially when applied to large-scale datasets. Although adversarial perturbations significantly enhance the robustness of the hidden information, the computational process may introduce additional burdens, potentially affecting overall efficiency. Therefore, optimizing the computational process while ensuring the quality of the stego-image and the precision of secret information extraction is crucial to reducing the demand on computational resources. Looking ahead, further research could explore how to improve the algorithm’s computational efficiency without affecting its robustness or embedding capacity. Moreover, the potential applications of AGASI can extend to secure communication and privacy protection, particularly in environments with high adversarial risks. Future work could also explore the opportunities for combining AGASI with other types of steganographic methods, such as multi-carrier or adaptive steganography, to further enhance its practical applications.

## Figures and Tables

**Table 1:** The qualities of ***C***, ***C′***, and ***C′_adv_*** under different perturbation intensities.

Image	Attack	Perturbation ε	PSNR	SSIM	SME
C $\;\mathrm{and}\;C^{\prime }$	None	None	32.924	0.944	0.047
C $\;\mathrm{and}\;{C^{\prime }}_{adv}$	FGSM	0.01	31.548	0.922	0.056
C $\;\mathrm{and}\;{C^{\prime }}_{adv}$	FGSM	0.02	28.984	0.876	0.063
C $\;\mathrm{and}\;{C^{\prime }}_{adv}$	FGSM	0.03	26.749	0.767	0.067
C $\;\mathrm{and}\;{C^{\prime }}_{adv}$	FGSM	0.04	24.451	0.731	0.071
C $\;\mathrm{and}\;{C^{\prime }}_{adv}$	FGSM	0.05	22.124	0.656	0.074
C $\;\mathrm{and}\;{C^{\prime }}_{adv}$	PGD	0.01	30.942	0.918	0.058
C $\;\mathrm{and}\;{C^{\prime }}_{adv}$	PGD	0.02	28.413	0.862	0.061
C $\;\mathrm{and}\;{C^{\prime }}_{adv}$	PGD	0.03	25.984	0.774	0.067
C $\;\mathrm{and}\;{C^{\prime }}_{adv}$	PGD	0.04	24.947	0.747	0.073
C $\;\mathrm{and}\;{C^{\prime }}_{adv}$	PGD	0.05	22.721	0.683	0.076

**Table 2:** The qualities of ***C***, ***C′***, and ***C′_adv’_*** under different disturbance intensities.

Image	Attack	Perturbation ε	PSNR	SSIM	SME
C $\;\mathrm{and}\;C^{\prime }$	None	None	32.924	0.944	0.047
C $\;\mathrm{and}\;{C^{\prime }}_{adv^{\prime }}$	FGSM	0.04	29.863	0.881	0.057
C $\;\mathrm{and}\;{C^{\prime }}_{adv^{\prime }}$	FGSM	0.08	28.718	0.854	0.063
C $\;\mathrm{and}\;{C^{\prime }}_{adv^{\prime }}$	FGSM	0.10	27.515	0.796	0.069
C $\;\mathrm{and}\;{C^{\prime }}_{adv^{\prime }}$	PGD	0.04	29.762	0.874	0.058
C $\;\mathrm{and}\;{C^{\prime }}_{adv^{\prime }}$	PGD	0.08	28.732	0.862	0.061
C $\;\mathrm{and}\;{C^{\prime }}_{adv^{\prime }}$	PGD	0.10	27.523	0.783	0.069

**Table 3:** The qualities of $S_{D},\;S_{adv}$, and $S^{\prime }$ under different disturbance intensities.

Image	Attack	Perturbation ε	PSNR	SSIM
S $\;\mathrm{and}\;S_{D}$	None	None	30.932	0.911
S $\;\mathrm{and}\;S_{adv}$	PGD	0.04	26.443	0.764
S $\;\mathrm{and}\;S^{\prime }$	PGD	0.04	29.146	0.887
S $\;\mathrm{and}\;S^{\prime }$	PGD	0.08	27.813	0.831
S $\;\mathrm{and}\;S^{\prime }$	PGD	0.10	27.237	0.804

**Table 4:** The effectiveness of AGASI in reducing the accuracy of Wide ResNet detection under different adversarial environments.

Perturbation	Wide ResNet (FGM)	Wide ResNet (PGD)
0.000	85.347	85.347
0.001	83.142	81.151
0.003	77.154	75.612
0.005	75.689	73.154
0.007	67.154	63.157
0.009	64.841	56.745
0.011	62.164	50.168
0.013	63.145	40.681
0.015	65.154	39.641
0.017	62.653	40.952
0.020	61.315	41.214
0.024	58.154	34.154
0.026	56.214	31.541
0.030	53.754	28.021

**Table 5:** Performance of AGASI secret information extraction with PGD attack.

Image	Attack	Perturbation ε	PSNR	SSIM
S $\;\mathrm{and}\;S_{D}$	None	None	31.022	0.951
S $\;\mathrm{and}\;S_{adv}$	PGD	0.004	26.043	0.764
S $\;\mathrm{and}\;S^{\prime }$	PGD	0.004	28.746	0.887
S $\;\mathrm{and}\;S^{\prime }$	PGD	0.006	27.813	0.843
S $\;\mathrm{and}\;S^{\prime }$	PGD	0.010	27.137	0.834

**Table 6:** Embedding capability of AGASI.

Method	Embedding Capacity (bpp)
LSB Matching Revisited [36]	1–2
Adaptive Steganography [37]	1–3
QIM [38]	2–4
AGASI	8

## Data Availability

The original contributions presented in this study are included in the article. Further inquiries can be directed to the corresponding author.
